# Complications of Resection Arthroplasty in Two-Stage Revision for the Treatment of Periprosthetic Hip Joint Infection

**DOI:** 10.3390/jcm8122224

**Published:** 2019-12-16

**Authors:** Irene K. Sigmund, Tobias Winkler, Nuri Önder, Carsten Perka, Nora Renz, Andrej Trampuz

**Affiliations:** 1 Center for Musculoskeletal Surgery (CMSC), Charite-Universitatsmedizin Berlin, Corporate Member of Freie Universität Berlin, Humboldt-Universität zu Berlin, and Berlin Institute of Health, Charitéplatz 1, D-10117 Berlin, Germany; tobias.winkler@charite.de (T.W.); carsten.perka@charite.de (C.P.); nora.renz@charite.de (N.R.); andrej.trampuz@charite.de (A.T.); 2 Medical University of Vienna, Department of Orthopaedics and Trauma Surgery, Spitalgasse 23, A-1090 Vienna, Austria; 3 Berlin Institute of Health Center for Regenerative Therapies (BCRT), Charite-Universitatsmedizin Berlin, Fohrer Str. 15, 13353 Berlin, Germany

**Keywords:** complication, resection arthroplasty, two-stage revision, reinfection, Girdlestone hip, periprosthetic joint infection, revision, hip

## Abstract

Little data is available regarding complications associated with resection arthroplasty in the treatment of hip periprosthetic joint infection (PJI). We assessed complications during and after two-stage revision using resection arthroplasty. In this retrospective study, 93 patients undergoing resection arthroplasty for hip PJI were included. Patients were assigned to a prosthesis-free interval of ≤10 weeks (group 1; 49 patients) or >10 weeks (group 2; 44 patients). The complication rates between groups were compared using the chi-squared test. The revision-free and infection-free survival was estimated using a Kaplan–Meier survival analysis. Seventy-one patients (76%) experienced at least one local complication (overall 146 complications). Common complications were blood loss during reimplantation (*n* = 25) or during explantation (*n* = 23), persistent infection (*n* = 16), leg length discrepancy (*n* = 13) and reinfection (*n* = 9). Patients in group 1 experienced less complications after reimplantation (*p* = 0.012). With increasing severity of acetabular bone defects, higher incidence of complications (*p* = 0.008), periprosthetic bone fractures (*p* = 0.05) and blood loss (*p* = 0.039) was observed. The infection-free survival rate at 24 months was 93.9% in group 1 and 85.9% in group 2. The indication for resection arthroplasty needs to be evaluated carefully, considering the high rate of complications and reduced mobility, particularly if longer prosthesis-free intervals are used.

## 1. Introduction

Periprosthetic joint infection (PJI) is one of the most challenging complications after total hip arthroplasty requiring comprehensive diagnostics, tailored surgical techniques, and an interdisciplinary approach to achieve high cure rates. Currently, there is no consensus on the optimal management of hip PJI. Two-stage revision protocols are the most widely used approach for infections after total hip arthroplasty with cure rates ranging from 67.7% to 96.7% [1,2,3,4]. An antibiotic-loaded polymethylmethacrylate (PMMA) bone cement spacer is commonly implanted in the prosthesis-free interval [5]. However, their use is not uniformly accepted, and several institutions prefer to use resection arthroplasty. Thus, the choice of cement spacer largely depends on surgical experience and tradition. Advantages of spacer use are local antimicrobial therapy, prevention of periarticular soft tissue contracture, and reduction of dead space [6,7], whereas the risk of peri-implant fractures, dislocations combined with acetabular damage, bacterial colonization of the spacer, and the risk of antimicrobial resistance emergence are commonly reported disadvantages [7,8,9]. Complications associated with cement spacer use are well reported in the literature [10]. 

During the original resection arthroplasty, first described 1928 for draining tuberculous hips and later in 1943 for severe pyogenic arthritis by Gathorne Robert Girdlestone, a muscle transposition into the acetabular fossa is performed as definitive treatment for hip infections [11,12]. Today, resection arthroplasty is recommended in difficult-to-treat infections caused by pathogens, for which no biofilm-active antibiotics and, hence, no eradication treatment exists [13]. Less data exists on the complications of resection arthroplasties used as a solution between stages before reimplantation. 

The aim of the current study is to assess the type and frequency of complications during and after two-stage revision performing resection arthroplasty during the interval. We evaluate local and systemic complications occurring during a two-stage revision involving revision arthroplasty, whether the incidence of complications differs in patients undergoing a shorter or longer prosthesis-free interval, and the overall revision-free and infection-free survival rate in patients treated by a two-stage revision with revision arthroplasty. In our institution, resection arthroplasty is the standard surgical treatment of hip PJI.

## 2. Materials and Methods

### 2.1. Study Design

This retrospective cohort study was conducted in a tertiary healthcare center specialized in septic surgery. Cases were identified from the patient-based PJI database and data obtained by reviewing electronic medical charts. The study protocol was reviewed and approved by the institutional ethics committee Berlin Charité-Universitätsmedizin Berlin (EA4/040/14) and was performed in accordance with the Declaration of Helsinki. Informed consent was waived due to the retrospective study design. 

### 2.2. Study Population 

Included were 93 patients with hip PJI, who had undergone a two-stage revision surgery with resection arthroplasty during prosthesis-free interval. Subjects treated at our institution from March 2006 to January 2014 were included. Patients with a prosthesis-free interval of ≤10 weeks were allocated to group 1 and patients with an interval of >10 weeks to group 2. The individual interval length was determined at the surgeon’s discretion, by the clinical course and operating room capacity. 

### 2.3. Data Collection 

Patients’ medical histories, radiographs, surgical records, microbiology and histopathology records, and laboratory analyses were reviewed. Information on demographics, preoperative acetabular and femoral bone deficiency (according to the Paprosky classification [14,15]), surgical treatment procedures and complications was collected. The following local complications were evaluated: Relevant blood loss (defined as requiring transfusion of erythrocyte concentrates), bone fissure, bone fracture, iatrogenic nerve damage, dislocation, microbiologically proven persistent infection (during prosthesis-free interval) or re-infection (after reimplantation), wound healing disorder, leg length discrepancy after reimplantation (>10 mm), aseptic loosening, massive haematoma requiring revision, ossification, pain (VAS ≥6 points), and bursitis trochanterica. Complications were divided into four groups according to their temporal appearance: Complications (i) during explantation, (ii) during the prosthesis-free interval, (iii) during reimplantation and (iv) after reimplantation. All systemic complications ocurring during the entire tretament period were assessed including allergic reaction to antibiotics, cardiovascular events, thromboembolic events, hepatic insufficiency, sepsis/systemic inflammatory response syndrome.

Patients with incomplete data sets or with follow-up of less than twelve months were excluded. PJI was diagnosed according to the 2017 proposed working criteria of the European Bone and Joint Infection Society (EBJIS) [16]. Treatment success was evaluated according to the Delphi international multidisciplinary consensus [17].

### 2.4. Surgical and Antimicrobial Treatment 

**Figure 1:**
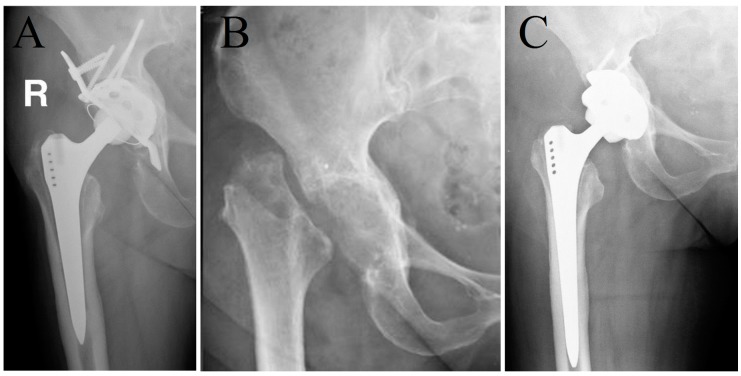
Anteroposterior radiograph of a 66-year old female patient with a periprosthetic joint infection of the right hip (**A**) before and (**B**) after the first stage (resection arthroplasty without spacer) and (**C**) after the second stage in a two-stage revision procedure for eradication of *Cutibacterium acnes*.

All included patients underwent a two-stage revision involving resection arthroplasty. At the first stage, all implants and foreign materials were removed (Figure 1). 

A thorough debridement was performed and at least three periprosthetic tissue samples were sent for microbiological analysis. To implant a new prosthesis at the second stage, the acetabular fossa remained empty during the prosthesis-free interval. After debridement and the positioning of drainage tubes, the wound was closed in layers.

Antimicrobial treatment was started after collecting the microbiological tissue samples or in the case of patients presenting with sepsis preoperatively after synovial aspiration. Antibiotics were administered intravenously for approximately two weeks after surgery, followed by oral antibiotics according to the susceptibility of the isolated pathogen(s) until reimplantation, if available [18]. The antibiotic treatment was given at the treating physician’s discretion and tailored to the isolated pathogen. No standardized antimicrobial protocol was followed.

During the prosthesis-free interval, toe-touch weight-bearing was allowed. If possible, patients were discharged after intravenous application of antibiotics (14 days). Wound controls were done every two weeks by their general practitioners. Fourteen days before reimplantation, patients had an appointment in our outpatient clinic for preoperative assessment.

Reimplantation was only performed when the wound was healed, soft tissues were ready for surgery, the general status of the patient was suitable, and C-reactive protein (CRP) was significantly decreased after explantation. At the second stage, again, at least three periprosthetic tissue samples were collected and sent for microbiological analysis. A thorough debridement followed by (uncemented or cemented according to the bone quality at the discretion of the operating surgeon) reimplantation of a hip prosthesis determined by the character of the bone defects were performed. In patients with small contained acetabular bone defects (Paprosky types 1 or 2) [15], a hemispherical uncemented component with or without screw placement was used. Depending on the bone defect, allograft impaction bone grafting was performed. If a high risk of dislocation was considered, a bipolar cup or constrained liner was used. Additional augments, and/or a reinforcement ring with a cemented liner/cup were used in hips in higher grade acetabular bone defects. A cementless non-modular stem was used in Paprosky type 1 and 2 femoral bone defects, and a cemented stem or an uncemented modular stem for larger femoral bone defects. In case of a persistent infection at the time of planned reimplantation surgery (discharging wound and/or increasing CRP without any other infection focus and/or local signs of infection), an additional revision with debridement was performed. 

The overall mean follow-up after the prosthesis reimplantation was 42.7 months (range: 13.1–104.6 months).

### 2.5. Demographics and Infection Characteristics 

One hundred and two patients underwent a two-stage exchange for hip PJI with resection arthroplasty during the evaluated period. After the exclusion of 10 patients because of insufficient follow-up, a total of 93 PJI of 92 patients (one patient with concomitant PJI of the right and left hip) were eligible for inclusion. The demographic data of the 93 analyzed cases are shown in Table 1. 

According to the duration of the prosthesis-free interval, 49 cases (53%) were allocated to group 1 and 44 cases (47%) to group 2. Statistically significant differences besides the interval duration between both study groups were only found regarding BMI and type 1 of Paprosky acetabular bone defect classification. The distribution of all causative microorganisms isolated during explantation and reimplantation is listed in Table 2. 

In 72 cases (77%), the pathogen was cultured during explantation. In 18 of these cases (25%), more than one microorganism caused the infection. During reimplantation surgery, cultures were positive in 17 cases (18%). In 5 of these cases (29%), polymicrobial infection was recorded. 

Sixteen patients (17.2%) had a persistent infection. Among those, 13 patients grew the same pathogen as isolated at the time of explantation, and, in 3 cases, a different microorganism was isolated. In one patient, *Staphylococcus simulans* (two positive tissue cultures, one positive synovial fluid culture) was isolated at the first stage and *Staphylococcus haemolyticus* (two positive synovial fluid cultures) at the second stage. In another patient, *Enterococcus faecium* (two positive synovial fluid cultures, two positive tissue cultures) was detected at the time of explantation, and *Staphylococcus aureus* and *Staphylococcus epidermidis* (one positive tissue culture and one positive synovial fluid culture each) at reimplantation. In the third patient, no bacterial growth was observed at the first stage, and *Staphylococcus aureus* (two positive tissue cultures) was isolated at the second stage. 

### 2.6. Statistical Analysis 

Continuous variables are expressed by median and range; categorical variables are described as absolute and relative frequencies (percentage). Student *t*-test and Fisher’s exact test were performed to compare metric and binary variables between both groups. A chi-squared test was used to compare the overall complication rate between both groups. Additionally, the revision-free and infection-free survival were calculated using Kaplan–Meier analysis. To test the equality of the survival distribution functions, a log-rank test was performed. All estimated parameters are reported with 95% confidence intervals. The significance level for all tests was 5% (*p* < 0.05). The software package XLSTATPM (version 2017; XLSTAT; Addinsoft, New York, NY, USA) was used for statistical analysis. 

## 3. Results

### 3.1. Local Complications and Their Temporal Appearance During the Two-Stage Revision 

Seventy-one patients (76%) had at least one local complication, and overall 146 complications occurred during the entire follow-up period. Table 3 summarizes local complications during different periods of the two-stage procedure. Common complications were relevant blood loss during reimplantation (*n* = 25), and during explantation (*n* = 23), persistent infection during prosthesis-free interval (*n* = 16), leg length discrepancy (*n* = 13), and reinfection (*n* = 9). 

During the prosthesis-free interval, two proximal femur fractures occurred and were stabilized intramedullary with cemented Steinmann pins. Another non-displaced proximal femur fracture detected during the interval was treated during reimplantation with cerclage wires. In 4 patients (4%), a wound-healing disorder (WHD) without the need for revision surgery was present.

Seven patients (8%) with acetabular Paprosky type 2 (*n* = 2) and type 3 (*n* = 5) or with femoral Paprosky type 1 (*n* = 1), type 2 (*n* = 2), type 3 (*n* = 1) and type 4 (*n* = 3) experienced a postoperative dislocation after a mean of 14.5 days (range: 8–26 days). While three patients had a successful closed reduction, four patients required revision surgery. In three patients, a dual mobility cup was implanted in which two had no further dislocation. One with a recurrent dislocation was treated with successful closed reduction and a temporary custom-made hip orthosis. No further dislocation was observed after the last intervention. Another patient with dislocation underwent successful stem exchange with reduction of antetorsion and increase in offset with no further dislocation. 

### 3.2. Systemic Complications 

Twenty-two (24%) patients experienced at least one systemic complication (Table 4), 12 (24%) in group 1 (12 complications) and 10 (23%) in group 2 (14 complications, *p* = 0.501). Thromboembolic events were more frequent in group 2 (*p* = 0.047).

### 3.3. Bone Defects and Consecutive Complications 

**Figure 2:**
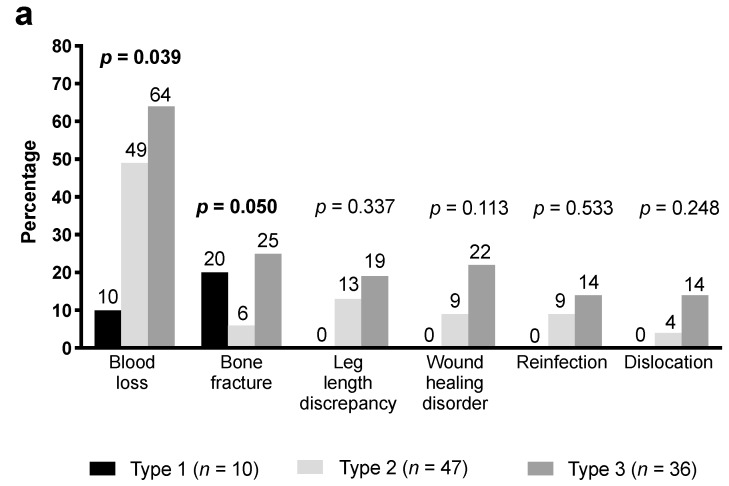

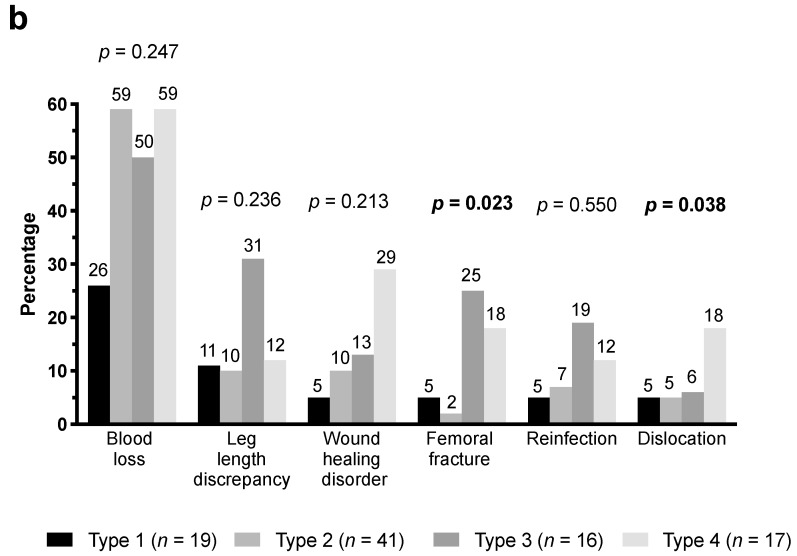
Frequency of complications in the different acetabular bone defect types according to Paprosky classification [15] (**a**) and femoral bone defect types according to Paprosky classification [14] (**b**). Number above the bars represent the frequency of the complication in the respective type.

A statistically significant difference of total number of complications comparing acetabular bone defect types (7 complications in 10 patients with type 1, 69 in 47 patients with type 2, and 70 in 36 patients with type 3; *p* = 0.008; Figure 2a) and femoral bone defect types (19 complications in 9 patients with type 1 (*n* = 19), 63 in 41 patients with type 2, 29 in 16 patients with type 3, and 35 in 17 patients with type 4; *p* = 0.043; Figure 2b) was observed. 

### 3.4. Comparison of Groups

**Figure 3:**
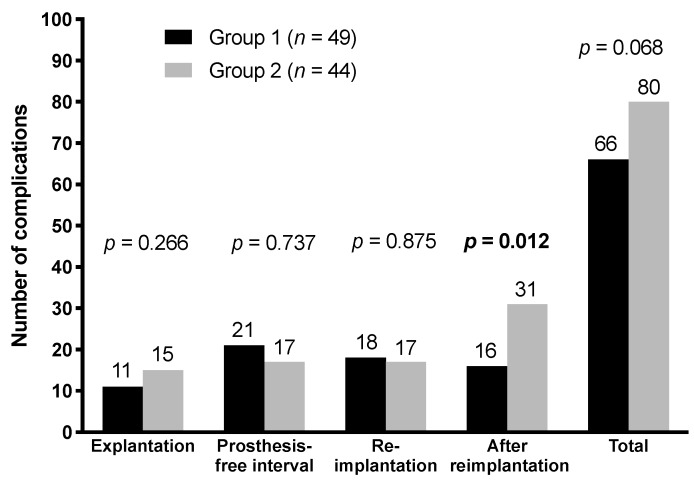
Complications during different periods of the two-stage procedure stratified according to the group.

In group 1, 66 local complications (45%) occurred during the whole study period, whereas 80 (55%) in group 2 (Figure 3). There were statistically significantly fewer complications after reimplantation of the hip prosthesis in group 1 (16 complications) compared with group 2 (31 complications) (*p* = 0.012). For no complication, a statistically significant difference between both groups was shown except for wound healing disorders after reimplantation (*p* = 0.009).

### 3.5. Revision-Free Survival 

**Figure 4:**
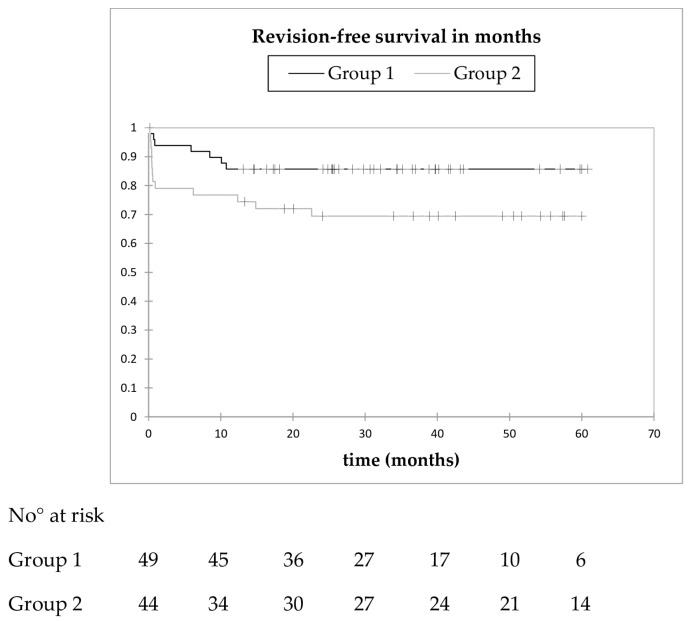
Kaplan–Meier revision-free survival curve as an endpoint in cases with an interval of ≤10 weeks (black) and an interval of >10 weeks (grey). No statistically significant difference between both groups was calculated (log-rank test, *p* = 0.058).

In group 1, the Kaplan–Meier analysis showed a revision-free survival probability of 91.8% (95% CI: 84.2–99.5) at six months, and 85.7% (95% CI: 75.9–95.5) at 12 months. After 11 months, no further revision was required. The mean revision-free survival time was 52.2 months (95% CI: 46.4–57.9). In group 2, revision-free survival probabilities of 81.4% (95% CI: 69.8–93.0) at six months, 74.4% (95% CI: 61.4–87.5) at 12 months, and 69.4% (95% CI: 55.6–83.3) at 24 months were calculated. All complications requiring revision surgery occurred within 2 years. The mean revision-free survival time was 41.3 months (95% CI: 33.6–48.9). There seems to be a higher revision-free survival rate in group 1 (Figure 4), but not at a statistically significant level (*p* = 0.058).

### 3.6. Infection-Free Survival 

**Figure 5:**
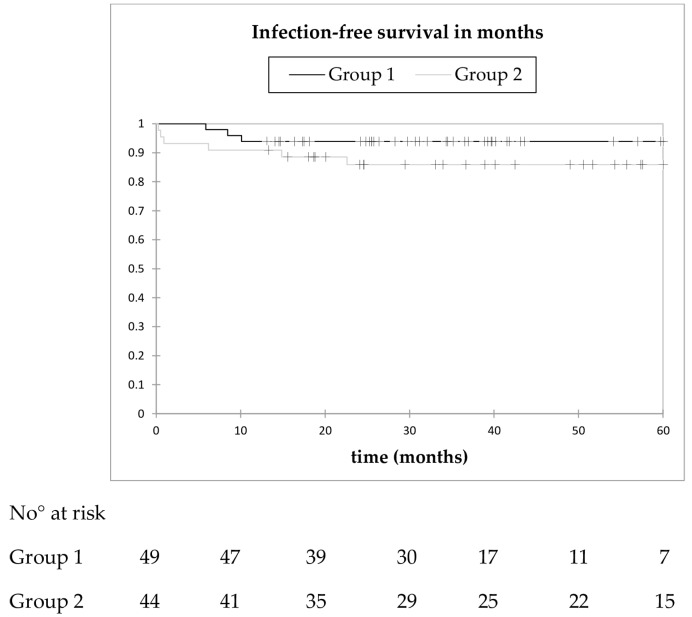
Kaplan–Meier infection-free survival curve in cases with an interval of ≤10 weeks (group 1, black) and an interval of >10 weeks (group 2, grey). No statistically significant difference between both groups was calculated (log-rank test, *p* = 0.223).

Overall, nine patients (10%) were revised because of infection after a mean time period of 7.8 months (range: 0.3–22.6). Of these, four (44%) were diagnosed as recurrent (one in group 1 and three in group 2) and five (56%) as new, haematogenous infections (two in group 1 and three in group 2). All were treated with at least one further surgical intervention. For the Kaplan–Meier analysis, all nine further infections were counted as reinfections (Figure 5). 

In group 1, all three infections occurred within 12 months, leading to an infection-free survival probability of 93.9% (95% CI: 87.2–100) at 12 months. The mean infection-free survival time was 72.1 months (95% CI: 66.5–77.7). Patients in group 2 showed an infection-free survival rate of 90.9% (95% CI: 82.4–99.4) at 12 months, and 85.9% (95% CI: 75.4–96.4) at 24 months. No further infection was diagnosed after 23 months. The mean infection-free survival time was 82.8 months (95% CI: 72.8–92.8). The Kaplan–Meier curves showed no significant difference between the two groups (*p* = 0.223).

## 4. Discussion

Few authors have systematically analysed local and systemic complications associated with resection arthroplasty for the treatment of hip PJI. Most common local complications in our study were relevant blood loss during reimplantation and during explantation, persistent infection during prosthesis-free interval, leg length discrepancy, and reinfection. 

While we had a low number of complications during the prosthesis-free interval in our study, spacer-related complications are commonly described in the interim period of a two-stage revision when using a PMMA bone cement spacer. Spacer related complication rates of 20–40% were reported which often require further surgery during the prosthesis-free interval [19,20,21]. Yang et al. showed a 45% (14/31) spacer related complication rate during the prosthesis-free interval in their cohort of 31 patients who underwent a two-stage revision with resection arthroplasty and implantation of cement spacers at the first stage. Six (19%) patients had a spacer dislocation, three (10%) a spacer fracture, four (13%) a femoral fracture, and one (3%) a spacer dislocation combined with a fracture. Faschingbauer et al. described a 20% complication rate associated with cement spacers [19]. Of 138 included patients, 12 (9%) suffered from a dislocation, further 12 (9%) had a spacer fracture, one (1%) had a femoral fracture, one a spacer fracture-dislocation, and one (1%) a spacer protrusion into the pelvis (1%). These high numbers of complications demonstrate the advantage of two-stage revision without a spacer. However, the most complained disadvantage of resection arthroplasty during the interim period is leg length discrepancy. The leg length discrepancy after conversion of resection arthroplasty into total hip arthroplasty occurs mainly due to soft tissue contracture and acetabular and femoral bone loss. Garcia-Rey et al. [22] showed greater leg length discrepancy in patients with conversion after a mean of 39 months compared to patients, who had revision surgery for aseptic loosening. Charlton et al [23] evaluated complications associated with reimplantation after Girdlestone arthroplasty in 44 patients. They demonstrated a leg length discrepancy in 50% of the study cohort with a mean discrepancy of 6.5 mm (5–30 mm) after placing a cement spacer into the acetabular bed during the interim period. In a study by Diemen et al. [10], where a cement spacer was also used during the interval, leg length discrepancy was shown in 8% of 136 cases with a mean discrepancy of 26 mm (range, 1.5–9 mm). In the current study, 14% patients showed leg length discrepancy with a median discrepancy of 15 mm. The patients in the group with shorter interval had less leg length discrepancies (8%) with a lower median discrepancy (13 mm) compared to the group with a larger prosthesis-free interval (20% and 20 mm, respectively). Therefore, with similar leg length discrepancy rates and median discrepancies, two-stage revision involving resection arthroplasty with a short interval was comparable with two-stage revision using a PMMA bone cement spacer during the interval. Other authors described more pronounced leg shortening in case of delayed reimplantation [24]. If an early reconstruction after resection arthroplasty is performed, results were similar to those in whom a spacer was used [10]. However, randomized studies comparing both treatment options with a short prosthesis-free interval are lacking. 

Dislocations occurred in 7.5% of patients (6% in group 1, 9% in group 2) within the first four weeks after reconstruction, which is in line with previous reports demonstrating a dislocation rate of 3%–11% in patients treated with resection arthroplasty [22,23,25] and comparable to the dislocation rate with cement spacer, which was described to be 1%–8% [10,22,26]. The probability of periprosthetic fracture after reimplantation in our study was 3.2%, which was similar to the reported incidence of 3.2% with resection arthroplasty and 4.3% with spacer use [22]. 

Importantly, patients with a shorter prosthesis-free interval had a lower local complication rate after reimplantation compared with patients treated with prosthesis-free interval >10 weeks, while the complications during the first and second stages showed no difference. Other authors also recommended early reimplantation to avoid soft tissue contracture and leg length discrepancy [27]. 

Of note, the extent of the acetabular and femoral bone defects influenced the complication rate. This could be explained by the more technically challenging reconstruction of a deficient bone stock to recreate the center of the hip rotation, which is more time consuming compared to hips with less deficiency [28]. In major acetabular bone defects, a higher overall complication rate was observed; for example, blood loss and bone fractures were more frequent in patients with larger bony deficiency. Dislocation, leg length discrepancy, nerve palsy, and wound healing disturbance were more common in type 2 and 3 acetabular bone defects. An interaction effect between the femoral bone defect types and the femoral fracture was also shown. Unsurprisingly, fractures were more frequent in patients with larger bony deficiency.

In our series, reinfection occurred in nine patients (9.7%) and was only present in patients with type 2 and 3 acetabular bone defects. Patients treated with a shorter prosthesis-free interval showed a trend towards lower reinfection rate (6%) compared with patients with a longer one (14%). These findings are in concordance with the reported reinfection rate ranging from 2.3 to 13.4% [10,23,25]. 

Regarding systemic complications, thromboembolic events were more often observed in patients with a longer prosthesis-free interval. Although low-molecular-weight heparin was given, four events were observed in the long interim period while none occurred in the short prosthesis-free interval group. A possible explanation could be the reduced mobilization during a long interval. However, each event occurred after reimplantation, when the patient was able to weight-bear again. In the study by Charlton et al, no thrombosis or embolism was reported [23]. Due to the controversial results, the impact of a long prosthesis-interval on thromboembolic events remains unclear. Further studies are needed to elucidate this problem. 

There are several limitations to this study. The present study did not include an evaluation of functional outcome. However, in the retrospective study by Marczak et al., the final Harris hip score (HHS) and visual analogue scale (VAS) score showed no difference between patients treated with a two-stage revision with or without spacer, while the final WOMAC (Western Ontario and McMaster Universities osteoarthritis index) score showed a better outcome in the spacer group [24]. The decision to use a spacer in this study was based on the preferences of the surgeon and local bone deficiency. Hence, resection arthroplasty was performed in patients with a severe bone deficiency, which could be a possible explanation for the significantly different WOMAC score. In the study by Hsieh et al. [6], the functional status of the patients (measured by using the Merle d’Aubigné and Postel hip score) was similar between patients treated with cement beads following resection arthroplasty and patients with a cement spacer during the prosthesis-free interval. Another limitation is the limited number of patients, making statistical comparisons between the groups difficult. Nevertheless, this retrospective series represents the largest single-center observation of two-stage revision involving Girdlestone hips during the prosthesis-free interval and still might be useful in solid decision making for orthopaedic surgeons.

## 5. Conclusions

In conclusion, complications of two-stage revision using resection arthroplasty were common, including relevant blood loss, leg length discrepancy, persistent infection, and reinfection. Nevertheless, the infection-free-survival was high (>85% after two years). Patients with shorter prosthesis-free intervals (≤10 weeks) experienced fewer complications after reimplantation. An increasing number of complications were seen with increasing acetabular defects. The indication for resection arthroplasty needs to be evaluated carefully, considering the high rate of complications and reduced mobility, particularly if longer prosthesis-free intervals are applied.

## Figures and Tables

**Table 1:** Demographic data of patients stratified in groups with a prosthesis-free interval of ≤10 weeks (group 1) and >10 weeks (group 2).

Variable	Group 1 (*n* = 49)	Group 2 (*n* = 44)	*p*-Value
Patient age, median (range)—years	75 (51–90)	77 (50–88)	0.790
Female sex	28 (57)	20 (45)	0.302
Body mass index (kg/m^2^)	29.2 (16.2–46.4)	25.7 (18.8–37)	0.014
ASA score	2.0 (2–3)	2.0 (1–3)	0.874
No. of previous surgeries	2 (0–8)	2 (0–10)	0.544
Acetabular bone defect (Paprosky [15])			
Type 1	9 (18)	1 (2)	0.017
Type 2	24 (49)	23 (52)	0.836
Type 3	16 (33)	20 (45)	0.286
Femoral bone defect (Paprosky [14])			
Type 1	14 (29)	5 (9)	0.070
Type 2	20 (41)	21 (48)	0.536
Type 3	8 (16)	8 (18)	1.000
Type 4	7 (14)	10 (23)	0.421
Prosthesis-free interval, median (range)—weeks	8.6 (1.0–10.0)	12.0 (10.1–115)	<0.0001
Type of prosthesis fixation			0.052
Cemented	13 (27%)	21 (48%)	
Uncemented	36 (73%)	23 (52%)	
Interval from reimplantation and first walk, median (range)—days	2.0 (1–10)	2.0 (1–6)	0.506

NOTE. The values are given as the number (percentage) of cases, if not otherwise indicated.

**Table 2:** Microbiological findings during explantation and reimplantation surgery among 93 cases.

Microorganism	Cases with Positive Microbiology at Explantation (*n* = 72)	Cases with Positive Microbiology at Reimplantation (*n* = 17)
Coagulase-negative staphylococci	53	17 ^1^
*Staphylococcus aureus*	11	2
*Cutibacterium acnes*	8	1
*Streptococcus* spp.	5	0
*Enterococcus* spp.	5	0
Gram-negative bacteria ^2^	5	0
Others ^3^	4	1

NOTE. The values are given as the number of cases. The sum exceeds the total of cases due to polymicrobial infections with multiple causative pathogens. ^1^ Of 19 positive tissue cultures with coagulase-negative staphylococci, in seven patients only one tissue sample showed growth but the growth was considered relevant as the patients were receiving antimicrobial treatment. ^2^ *Escherichia coli (n* = 2), *Salmonella* spp. *(n* = 1), *Achromobacter xylosoxidans* (*n* = 1), *Pseudomonas putida* (*n* = 1). ^3^ During explantation: *Corynebacterium* spp. (*n* = 1), *Granulicatella adiacens* (*n* = 1), *Actinomyces neuii* (*n* = 1), *Lactobacillus* spp. (*n* = 1), during reimplantation: *Dermabacter hominis* (*n* = 1).

**Table 3:** Local complications occurring during different periods of the two-stage procedure in the group with an interval of ≤10 weeks (group 1) and >10 weeks (group 2).

Complication	All Cases (*n* = 93)	Group 1 (*n* = 49)	Group 2 (*n* = 44)	*p*-Value
**At explantation**				
Blood loss ^1^	22 (24)	9 (18)	13 (30)	0.230
Bone fracture ^2^	3 (3)	2 (4)	1 (2)	1.000
Nerve palsy ^3^	1 (1)	0 (0)	1 (2)	0.473
Total	26 (28)	11 (22)	15 (34)	0.266
**During resection arthroplasty**				
Persistent infection	16 (17)	8 (16)	8 (18)	1.000
Wound healing disorder	6 (6)	2 (4)	4 (9)	0.417
Bone fracture ^4^	3 (3)	2 (4)	1 (2)	1.000
Others ^5^	13 (14)	9 (18)	4 (9)	0.268
Total	38 (41)	21 (43)	17 (39)	0.737
**At reimplantation**				
Blood loss ^6^	25 (27)	13 (27)	12 (27)	1.000
Bone fracture ^7^	5 (5)	3 (6)	2 (5)	1.000
New infection	3 (3)	1 (2)	2 (5)	0.601
Nerve palsy ^8^ (reversible)	2 (2)	1 (2)	1 (2)	1.000
Total	35 (38)	18 (37)	17 (39)	0.875
**After Reimplantation**				
Leg length discrepancy ^9^	13 (14)	4 (8)	9 (20)	0.134
Reinfection	9 (10)	3 (6)	6 (14)	0.299
Dislocation	7 (8)	3 (6)	4 (9)	0.704
Wound healing disturbance ^10^	6 (7)	0 (0)	6 (14)	0.009
Bone fracture ^11^	3 (3)	2 (4)	1 (2)	1.000
Aseptic loosening ^12^	2 (2)	0 (0)	2 (5)	0.226
Others ^13^	7 (8)	4 (8)	3 (7)	0.688
Total	47 (51)	16 (33)	31 (70)	0.012
**Total**	**146**	**66**	**80**	**0.068**

NOTE. The values are given as the number (percentage) of cases. ^1^ Requiring transfusion of 2–9 (median 2) erythrocyte concentrates. ^2^ Including 2 fissures and 1 fracture of the proximal femur stabilized with additional cerclage wires. ^3^ Temporary palsy of the peroneal nerve (full recovery within 6 weeks). ^4^ Including 2 proximal femur fractures stabilized intramedullary with cemented Steinmann pins and 1 non-displaced proximal femur fracture treated with cerclage wires. ^5^ Including heterotopic ossification (*n* = 8), haematoma (*n* = 1), pain with VAS ≥6 points (*n* = 2), and bursitis trochanterica (*n* = 2). ^6^ Requiring transfusion of 2–14 (median 2) erythrocyte concentrates. ^7^ Including 4 fractures of the proximal femur stabilized with additional cerclage wires and 1 acetabulum fracture reconstructed with an acetabular reinforcement ring and cemented cup. ^8^ Temporary palsy of the femoral nerve with full recovery within 8 weeks (in the patient with the acetabulum fracture). ^9^ Median leg length discrepancy of 15 mm (range: 10–35 mm); in group 1, four (8%) patients had a median leg length discrepancy of 13 mm (range: 10–20 mm) and in group 2, nine (20%) patients with a median leg length discrepancy of 20 mm (range: 10–35 mm) (*p* = 0.134). ^10^ Two patients required revision surgery. ^11^ One proximal femur fracture (treated with a fracture fixation plate and cerclage wires), one acetabular fracture (navigated percutaneous screw fixation), and one ischial tuberosity avulsion fracture (treated conservatively). ^12^ Evaluated at follow-up visits (after 49.7 and 69.0 months), no pain, no required revision. ^13^ Heterotopic ossification (*n* = 1), haematoma (with required revision surgery; (*n* = 3)), pain with VAS ≥6 points (*n* = 3).

**Table 4:** Systemic complications occurring during different periods of the two-stage procedure in the group with an interval of ≤10 weeks (group 1) and >10 weeks (group 2).

Complications	All Cases (*n* = 93)	Group 1 (*n* = 49)	Group 2 (*n* = 44)	*p*-Value
Allergic reaction to antibiotics	6	5	1	0.120
Cardiovascular events ^1^	6	5	1	0.208
Thromboembolic events ^2^	4	0	4	0.047
Hepatic insufficiency *	2	0	2	0.131
Sepsis/Systemic inflammatory response syndrome *	2	0	2	0.131
Others ^3^	6	2	4	0.417
**Total**	**26**	**12**	**14**	**0.501**

NOTE. * denotes complications occurring after explantation. ^1^ Including atrial fibrillation (*n* = 2), supraventricular tachycardia (*n* = 1), acute ischemic stroke (*n* = 1), myocardial infarction (*n* = 1) and endocarditis (*n* = 1). ^2^ Including deep vein thrombosis (*n* = 3) and pulmonary embolism (*n* = 1). ^3^ Sacral pressure sore (*n* = 1), pneumothorax (*n* = 1), paralytic ileus (*n* = 1), hyponatremia (*n* = 1), cholecystolithiasis* (*n* = 1) and acute kidney failure* (*n* = 1).
